# Optimal designs of the side sensitive synthetic chart for the coefficient of variation based on the median run length and expected median run length

**DOI:** 10.1371/journal.pone.0255366

**Published:** 2021-07-30

**Authors:** Waie Chung Yeong, Ping Yin Lee, Sok Li Lim, Peh Sang Ng, Khai Wah Khaw

**Affiliations:** 1 School of Mathematical Sciences, Sunway University, Petaling Jaya, Malaysia; 2 Institute of Mathematical Sciences, Faculty of Science, Universiti Malaya, Kuala Lumpur, Malaysia; 3 Department of Physical and Mathematical Science, Faculty of Science, Universiti Tunku Abdul Rahman, Perak, Malaysia; 4 School of Management, Universiti Sains Malaysia, Penang, Malaysia; Universidade de Vigo, SPAIN

## Abstract

The side sensitive synthetic chart was proposed to improve the performance of the synthetic chart to monitor shifts in the coefficient of variation (*γ*), by incorporating the side sensitivity feature where successive non-conforming samples must fall on the same side of the control limits. The existing side sensitive synthetic- *γ* chart is only evaluated in terms of the average run length (*ARL*) and expected average run length (*EARL*). However, the run length distribution is skewed to the right, hence the actual performance of the chart may be frequently different from what is shown by the *ARL* and *EARL*. This paper evaluates the entire run length distribution by studying the percentiles of the run length distribution. It is shown that false alarms frequently happen much earlier than the in-control *ARL* (*ARL*_0_), and small shifts are often detected earlier compared to the *ARL*_1_. Subsequently, this paper proposes an alternative design based on the median run length (*MRL*) and expected median run length (*EMRL*). The optimal design based on the *MRL* shows smaller out-of-control *MRL* (*MRL*_1_), which shows a quicker detection of the out-of-control condition, compared to the existing design, while the results from the optimal design based on the *EMRL* is similar to that of the existing designs. Comparisons with the synthetic-*γ* chart without side sensitivity shows that side sensitivity reduces the median number of samples required to detect a shift and reduces the variability in the run length. Finally, the proposed designs are implemented on an actual industrial example.

## 1. Introduction

Control charts are useful tools to detect the presence of assignable cause(s) which results in an out-of-control process. By convention, control charts monitor the process mean (*μ*) and/or standard deviation (*σ*), where shifts in *μ* and/or *σ* shows the presence of assignable cause(s). For example, readers can refer to the studies by Aslam et al. [1], Riaz et al. [2], Abujiya et al. [3], and many others.

However, conventional charts in the preceding paragraph could not be used to monitor all types of processes. This is because not all processes have a constant *μ*. Furthermore, *σ* may change according to *μ*. For such processes, monitoring *μ* and/or *σ* will result in dubious conclusions, since shifts in *μ* and/or *σ* does not mean that the process is out-of-control. Kang et al. [4] proposed to monitor such processes through the coefficient of variation (*γ*), where $\gamma =\frac{\sigma }{\mu }$. Assignable cause(s) is detected when there is a shift in the linear relationship between *σ* and *μ*. Kang et al. [4] monitored *γ* only through the present sample, hence the chart is not sensitive towards small and moderate shifts in *γ*.

Since the first *γ* chart was proposed by Kang et al. [4], numerous new and better charts are proposed to monitor *γ*. Hong et al. [5] was the first to propose an Exponentially Weighted Moving Average (EWMA) *γ* chart, followed by numerous improvements on the EWMA chart by Castagliola et al. [6], Zhang et al. [7] and Zhang et al. [8]. Besides the EWMA chart, other charts proposed to monitor *γ* include the run rules chart [9], side sensitive group runs (SSGR) chart [10] and run sum chart [11]. *γ* charts with variable charting parameters was first proposed by Castagliola et al. [12], who varied the sampling interval. Subsequently, the variable sample size *γ* chart [13, 14], variable sample size and sampling interval *γ* chart [15], variable sampling interval EWMA *γ* chart [16], variable parameters *γ* chart [17], and variable sample size EWMA *γ* chart [18] are proposed. Charts monitoring *γ* that is designed by considering measurement errors are also proposed Yeong et al. [19], Tran et al. [20], Tran et al. [21]and Saha et al. [22].

Calzada and Scariano [23] proposed the synthetic chart to monitor *γ*. The synthetic chart waits until the second point to fall outside the control limits before deciding whether the process is in-control or out-of-control. Samples that fall inside the control limits are called conforming samples, while those that fall outside the control limits are called non-conforming samples. If two successive non-conforming samples happen too close to each other, the process is out-of-control. Calzada and Scariano [23] showed that the synthetic-*γ* chart outperforms the Shewhart-*γ* chart for all shift sizes, but does not outperform the Exponentially Weighted Moving Average (EWMA)-*γ* chart, proposed by Castagliola et al. [6], for small and moderate shift sizes.

The synthetic-*γ* chart does not differentiate between non-conforming samples that fall outside the upper control limit (*UCL*) and those that fall below the lower control limit (*LCL*). Hence, as long as the samples fall either outside the *UCL* or below the *LCL*, they are considered to be non-conforming samples. Successive non-conforming samples do not have to fall on the same side of the control limits. In other words, if the first non-conforming sample falls above the *UCL*, the next non-conforming sample can either fall above the *UCL* or below the *LCL*.

Subsequently, Yeong et al. [24] proposed the side sensitive synthetic-*γ* chart. In the side sensitive synthetic-*γ* chart, successive non-conforming samples must fall on the same side of the control limits. For example, if the first non-conforming sample falls above the *UCL*, the next non-conforming sample must also fall above the *UCL*. Samples that fall below the *LCL* are not considered to be non-conforming in this case. The side sensitive synthetic-*γ* chart is shown to result in a significant improvement over the performance of the synthetic-*γ* chart proposed by Calzada and Scariano [23]. Furthermore, unlike the synthetic-*γ* chart, the side sensitive synthetic-*γ* chart showed comparable or better performance than the EWMA-*γ* chart for most shift sizes, except for very small shift sizes.

In Yeong et al. [24], the performance of the side sensitive synthetic-*γ* chart is evaluated only in terms of the average run length (*ARL*) and expected average run length (*EARL*). There are two types of *ARL*, the in-control *ARL* (*ARL*_0_), which is the average number of samples taken until a false alarm occurs, and the out-of-control *ARL* (*ARL*_1_), which is the average number of samples taken until a shift of a specific magnitude is detected. The *ARL*_1_ requires the shift size to be specified. It is difficult to specify the shift size in a lot of practical scenarios, hence the side sensitive synthetic-*γ* chart is also evaluated in terms of the *EARL*, which is the expected average number of samples taken to detect a shift that is specified as a range of values.

Evaluating a chart’s performance based solely on the *ARL* might lead to confusion on the actual performance of the chart [25]. This is because, for in-control processes or out-of-control processes with small shift sizes, the run length distribution is highly skewed to the right [26]. For a right-skewed distribution, the median is smaller than the mean, hence the median run length (*MRL*) will be smaller than the *ARL*. When the in-control *MRL* (*MRL*_0_) is smaller than the *ARL*_0_, more than 50% of the in-control run lengths will be smaller than the *ARL*_0_. In other words, for more than 50% of the time, the false alarm will happen before what is indicated by the *ARL*_0_. When this happens, practitioners would have reduced confidence towards the *ARL*_0_ as a performance measure. Hence, in this paper, the performance of the side sensitive synthetic-*γ* chart is evaluated based on its’ entire run length distribution to have a clearer idea of its’ actual performance. An analysis of the in-control and out-of-control percentiles (for known shift sizes) and expected percentiles (for unknown shift sizes) will be conducted.

The optimal charting parameters of the existing side sensitive synthetic-*γ* chart is obtained based on two designs, where the first design optimizes the *ARL*_1_ (for known shift sizes) while the second optimizes the *EARL* (for unknown shift size). For both designs, constraints in *ARL*_0_ needs to be satisfied. However, since the run length distribution is skewed to the right (for in-control and out-of-control run lengths for small shift sizes), alternative designs are proposed to obtain the optimal charting parameters of the side sensitive synthetic-*γ* chart. In this paper, alternative designs are proposed where the optimal charting parameters are obtained to minimize the out-of-control *MRL* (*MRL*_1_) and the expected *MRL* (*EMRL*), subject to constraints in the *MRL*_0_. This is because the *MRL* is a more accurate measure of performance when the run length is skewed.

Designs based on the *MRL* are available for several charts in the literature, among some of the more recent ones are studies on the *MRL* performance of the synthetic $\bar{X}$ chart by Hu et al. [27], optimal designs based on the *MRL* and/or *EMRL* for the one-sided exponential CUSUM chart [28], one-sided exponential EWMA chart [29], EWMA-*γ* chart [30], EWMA $\bar{X}$ chart [31], variable sample size $\bar{X}$ chart [32], synthetic np chart [33], multivariate synthetic |*S*| chart [34], double sampling $\bar{X}$ chart [35], and many others. However, the design of the side sensitive synthetic-*γ* chart is not available in the literature. This paper will fill this gap.

The rest of the paper is organized as follows. The next section gives an overview of the side sensitive synthetic-*γ* chart, as well as the formulae to evaluate the *ARL*, standard deviation of the run length (*SDRL*), *EARL* and the percentiles of the run length distribution. Section 3 analyses the percentiles of the run length distribution by adopting the optimal charting parameters by Yeong et al. [24]. Subsequently, Section 4 proposes the algorithms to obtain the optimal charting parameters based on the *MRL*_1_ and *EMRL*, and illustrates the optimal charting parameters, *MRL*_1_ and *EMRL* based on several numerical examples. This is followed by a comparison with the synthetic-*γ* chart without side sensitivity in Section 5, and the implementation of the proposed designs on an actual industrial example in Section 6. Finally, some concluding remarks are given in Section 7.

## 2. Side sensitive synthetic-*γ* chart

The synthetic-*γ* chart works by waiting until the second sample to fall outside the control limits before deciding whether the process is in-control or out-of-control. For the synthetic-*γ* chart without the side sensitivity feature, when the sample coefficient of variation $\left(\hat{\gamma }\right)$ falls within the *UCL* and *LCL*, the sample is a conforming sample, while if $\hat{\gamma }>UCL$ or $\hat{\gamma }<LCL$, the sample is a non-conforming sample. The number of conforming samples between two successive non-conforming samples (including the ending non-conforming sample) is referred to as the conforming run length (*CRL*). For example, when there are three conforming samples between two successive non-conforming samples, the *CRL* = 4. When *CRL*≤*L*, where *L* is a pre-determined threshold set by the practitioner, the synthetic-*γ* chart will produce an out-of-control signal. To give the synthetic-*γ* chart a head-start, the first *CRL* counts the number of conforming samples until the first non-conforming sample appears.

**Fig 1:**
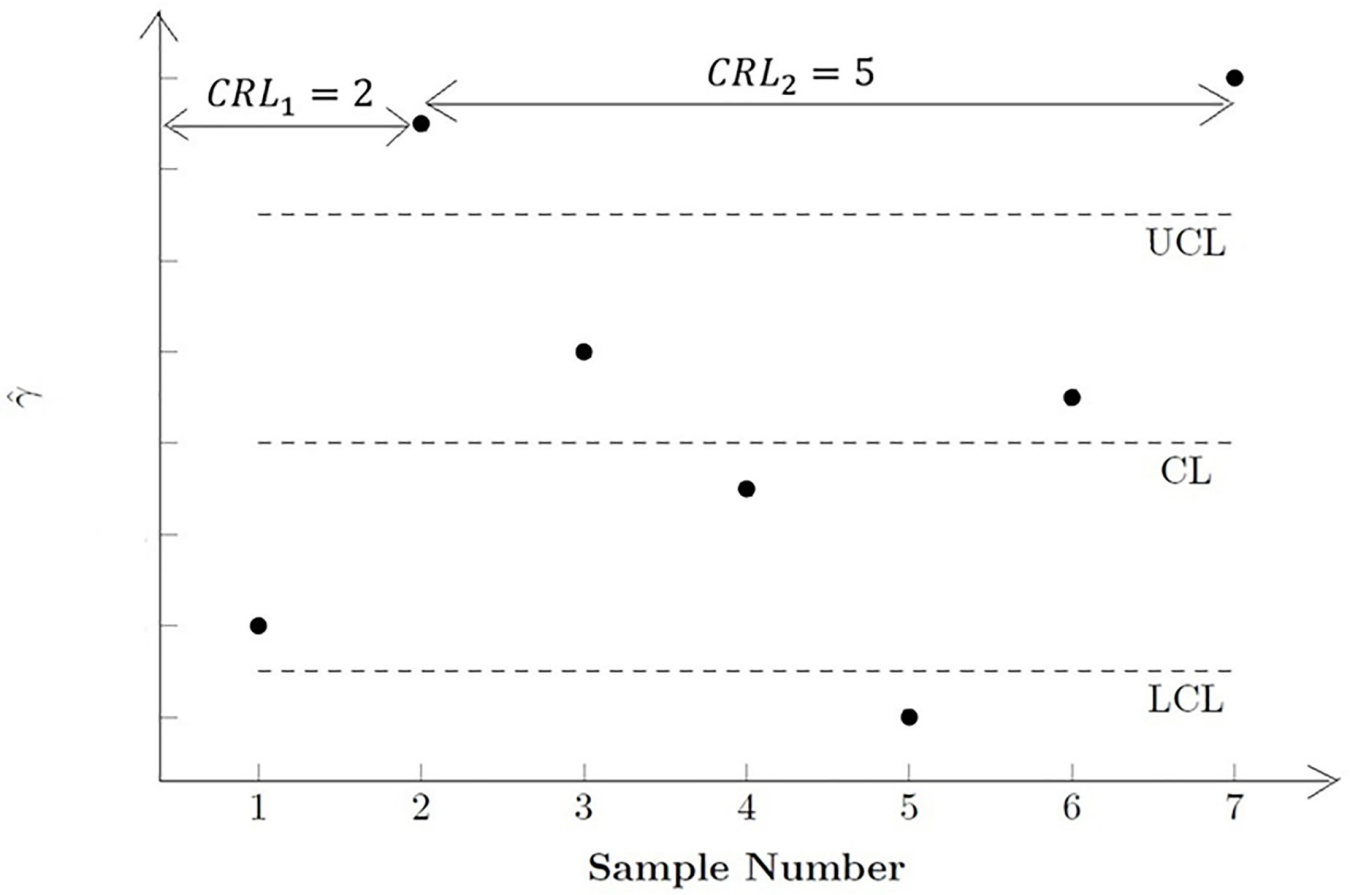
The *CRL* sub-chart of the side-sensitive synthetic-*γ* chart.

The main difference between the side sensitive synthetic-*γ* chart and the synthetic-*γ* chart is that successive non-conforming samples must fall on the same side of the control limits. For example, by referring to the *CRL* sub-chart of the side sensitive synthetic-*γ* chart in Fig 1, since the first non-conforming sample (Sample 2) falls above the *UCL*, only samples that fall above the *UCL* are considered to be non-conforming samples. Although Sample 5 falls outside the control limits, it is still a conforming sample as it falls below the *LCL*. The next non-conforming only occurs in Sample 7. Hence, the *CRL* = 5.

The *LCL* and *UCL* of the side sensitive synthetic-*γ* chart are computed as

$$LCL=\mu_{0}(\hat{\gamma })-K\sigma_{0}(\hat{\gamma }),$$ (1)

and

$$UCL=\mu_{0}(\hat{\gamma })+K\sigma_{0}(\hat{\gamma }),$$ (2)

where *K* represents the control limit coefficient, while $\mu_{0}(\hat{\gamma })$ and $\sigma_{0}(\hat{\gamma })$ are the in-control mean and standard deviation of $\hat{\gamma }$, respectively. Although $\mu_{0}(\hat{\gamma })$ and $\sigma_{0}(\hat{\gamma })$ have no closed forms, the following approximations by Reh and Scheffler [36]will be used, i.e.

$$\mu_{0}(\hat{\gamma })\approx \gamma_{0}[1+\frac{1}{n}(\gamma_{0}^{2}-\frac{1}{4})+\frac{1}{n_{}^{2}}(3\gamma_{0}^{4}-\frac{\gamma_{0}^{2}}{4}-\frac{7}{32})+\frac{1}{n_{}^{3}}(15\gamma_{0}^{6}-\frac{3\gamma_{0}^{4}}{4}-\frac{7\gamma_{0}^{2}}{32}-\frac{19}{128})]$$ (3)

and

$$\sigma_{0}(\hat{\gamma })\approx \gamma_{0}\sqrt{\frac{1}{n}(\gamma_{0}^{2}+\frac{1}{2})+\frac{1}{n_{}^{2}}(8\gamma_{0}^{4}+\gamma_{0}^{2}+\frac{3}{8})+\frac{1}{n_{}^{3}}(69\gamma_{0}^{6}+\frac{7\gamma_{0}^{4}}{2}+\frac{3\gamma_{0}^{2}}{4}+\frac{3}{16})},$$ (4)

where *γ*_0_ and *n* are the in-control *γ* and sample size, respectively.

A Markov chain approach is adopted to evaluate the *ARL*, *SDRL*, *EARL* and the percentiles of the run length distribution. The formulae to evaluate the *ARL*, *SDRL* and *EARL* is shown in Yeong et al. [24] and is reproduced in S1 Appendix for ease of reference.

To study the percentiles of the run length distribution, the probability mass function (p.m.f.) and cumulative distribution function (c.d.f.) of the run length needs to be obtained. The p.m.f. and c.d.f. of the run length for the side sensitive synthetic-*γ* chart can be computed as [37]

$$f_{RL}(l)=P(RL=l)=q^{T}(Q^{l-1})r,$$ (5)

and

$$F_{RL}(l)=P(RL\leq l)=1-q^{T}(Q^{l-1})1,$$ (6)

where *l*∈{1,2,3,…}, **q** and **1** are as defined in Equations (A5) and (A6), while **Q** and **r** are as defined in Equation (A1).

The (100*θ*)^th^ percentile of the run length distribution of the side sensitive synthetic-*γ* chart can be obtained from Eq (6) by searching for *l*_*θ*_ such that [26]

$$P(RL\leq l_{\theta }-1)\leq \theta \;\mathrm{and}\;P(RL\leq l_{\theta })>\theta ,$$ (7)

where 0<*θ*<1. For example, the *MRL*, which is the 50^th^ percentile of the run length, can be obtained from Eq (7) by setting *θ* = 0.5. The in-control percentiles can be obtained by setting *γ* = *γ*_0_ when computing the transition probabilities of **Q** through Equations (A2) to (A4), while the out-of-control percentiles are obtained by setting *γ* = *τγ*_0_, where *τ* is the shift size.

The percentiles of the run length can only be obtained if *τ* can be known in advance. However, practitioners usually find it difficult to specify *τ* due to insufficient data. Furthermore, *τ* may not be deterministic and may vary according to some unknown stochastic model [6]. Under such conditions, the expected percentiles of the run-length distribution, *E*(*l*_*θ*_) are evaluated instead. *E*(*l*_*θ*_) does not require *τ* to be specified as a specific value. Instead, *τ* only needs to be specified as a range of possible values, (*τ*_min_,*τ*_max_). *E*(*l*_*θ*_) can be computed as follows:

$$E(l_{\theta })=\int_{\tau_{\min}}^{\tau_{\max}}f_{\tau }(\tau )l_{\theta }(\tau )d\tau ,$$ (8)

where *f*_*τ*_(*τ*) is the probability density function (p.d.f.) of *τ*. It is assumed that *f*_*τ*_(*τ*) is a uniformly distributed continuous random variable over the interval (*τ*_min_,*τ*_max_) [6]. The integral in Eq (8) is approximated using the Gauss-Legendre quadrature [38].

## 3. Analysis of the percentiles of the run length distribution

In this section, the percentiles of the run length distribution are analysed based on the optimal charting parameters by Yeong et al. [24]. Yeong et al. [24] adopted two types of designs. In the first design, the optimal charting parameters are obtained to minimize *ARL*_1_, subject to constraints in the *ARL*_0_, while the second design obtains the optimal charting parameters to minimize the *EARL*, subject to the same constraints in *ARL*_0_. For ease of reference, the first and second designs are referred to as *ARL*-based and *EARL*-based designs, respectively. Both the in-control and out-of-control run length percentiles are analysed in this section.

Table 1 shows the optimal charting parameters (*L*, *LCL* and *UCL*), the *ARL*_1_ and *SDRL*_1_ of the *ARL*-based design of the side sensitive synthetic-*γ* chart for *γ*_0_ = 0.05,*n*∈{5,7,10,15} and *τ*∈{1.1,1.2,1.3,1.5,2.0}. Note that *τ*>1 indicates an upward shift in *γ*_0_, while *τ*<1 indicates a downward shift. Practitioners are often more interested in detecting an upward shift, since an upward shift results in a larger $\frac{\sigma }{\mu }$ ratio which shows that there is increased variability in the process due to a relatively large *σ* compared to *μ*. The increased variability is usually due to the presence of assignable cause(s). It is important to detect the presence of such assignable cause(s) so that they can be removed to reduce variability in the process. Comparatively, detecting a downward shift is less crucial as a downward shift shows decreased variability. Thus, this paper only considers *τ*>1.

**Table 1:** Optimal charting parameters and the corresponding *ARL*_1_ and *SDRL*_1_ values for the *ARL*-based side sensitive synthetic-*γ* chart for *γ*_0_ = 0.05, *n*∈{5,7,10,15} and *τ*∈{1.1,1.2,1.3,1.5,2.0}.

*τ*	*L*	*LCL*	*UCL*	*ARL*_1_	*SDRL*_1_
	*n* = 5				
1.1	42	0.0017	0.0924	64.74	84.69
1.2	23	0.0039	0.0902	21.35	27.11
1.3	15	0.0055	0.0885	10.18	12.25
1.5	8	0.0080	0.0860	4.18	4.44
2.0	4	0.0109	0.0832	1.72	1.27
*τ*	*L*	*LCL*	*UCL*	*ARL*_1_	*SDRL*_1_
	*n* = 7				
1.1	37	0.0116	0.0843	52.13	67.97
1.2	19	0.0136	0.0824	15.75	19.66
1.3	12	0.0150	0.0810	7.30	8.47
1.5	7	0.0166	0.0794	3.02	2.89
2.0	3	0.0192	0.0767	1.38	0.82
*τ*	*L*	*LCL*	*UCL*	*ARL*_1_	*SDRL*_1_
	*n* = 10				
1.1	35	0.0193	0.0780	41.27	53.44
1.2	16	0.0210	0.0762	11.43	13.91
1.3	10	0.0221	0.0752	5.19	5.68
1.5	5	0.0238	0.0735	2.22	1.91
2.0	3	0.0250	0.0723	1.17	0.47
*τ*	*L*	*LCL*	*UCL*	*ARL*_1_	*SDRL*_1_
	*n* = 15				
1.1	31	0.0259	0.0724	31.15	39.93
1.2	13	0.0274	0.0709	7.84	9.14
1.3	7	0.0285	0.0697	3.53	3.61
1.5	4	0.0296	0.0687	1.63	1.13
2.0	2	0.0309	0.0673	1.05	0.24

The optimal charting parameters in Table 1 are obtained to minimize the *ARL*_1_, subject to *ARL*_0_ = 370.4. For example, for *n* = 5 and *τ* = 1.1, adopting the charting parameters (*L*,*LCL*,*UCL*) = (42,0.0017,0.0924) will result in the smallest *ARL*_1_, while maintaining the *ARL*_0_ as 370.4. The (*ARL*_1_,*SDRL*_1_) = 64.74,84.69) when these optimal charting parameters are adopted.

To get a better picture on the actual run length performance of the *ARL*-based side sensitive synthetic-*γ* chart, the entire in-control and out-of-control run length distribution is analyzed. Table 2 shows the 5^th^ until 95^th^ in-control percentiles of the side sensitive synthetic-*γ* chart, while Table 3 shows the corresponding out-of-control percentiles. The percentiles are obtained by substituting the optimal charting parameters in Table 1, together with the corresponding *γ*_0_, *n* and *τ*, into Eq (7).

**Table 2:** In-control percentiles of the run length distribution for the *ARL*-based side sensitive synthetic-*γ* chart for *γ*_0_ = 0.05, *n*∈{5,7,10,15} and *τ*∈{1.1,1.2,1.3,1.5,2.0}.

	5^th^	10^th^	20^th^	30^th^	40^th^	50^th^	60^th^	70^th^	80^th^	90^th^	95^th^
*τ*	*n* = 5										
1.1	6	13	26	41	125	211	316	451	641	967	1293
1.2	5	10	20	74	143	225	325	454	636	947	1258
1.3	4	8	26	84	150	229	325	448	623	921	1220
1.5	3	6	41	97	161	237	329	449	618	906	1194
2.0	2	4	54	108	171	244	335	451	615	895	1176
*τ*	*n* = 7										
1.1	6	12	24	55	128	211	313	445	631	948	1266
1.2	5	9	18	80	148	228	326	453	631	936	1242
1.3	4	7	34	90	156	233	328	450	622	917	1211
1.5	3	6	46	102	166	242	336	456	625	914	1203
2.0	2	12	59	111	172	244	332	446	606	880	1154
*τ*	*n* = 10										
1.1	6	12	24	63	133	216	318	449	634	950	1266
1.2	4	8	27	84	150	228	323	446	619	915	1210
1.3	4	7	41	98	164	242	337	460	633	928	1224
1.5	3	5	53	107	170	243	334	450	614	895	1175
2.0	2	14	62	116	178	251	341	457	621	900	1180
*τ*	*n* = 15										
1.1	6	12	24	72	141	224	325	455	638	951	1265
1.2	4	8	37	94	160	238	334	457	630	927	1223
1.3	3	6	48	102	165	239	329	445	609	890	1170
1.5	3	11	58	111	173	246	335	450	612	888	1165
2.0	2	20	65	117	177	247	334	446	603	872	1141

**Table 3:** Out-of-control percentiles of the run length distribution for the *ARL*-based side sensitive synthetic-*γ* chart for *γ*_0_ = 0.05, *n*∈{5,7,10,15} and *τ*∈{1.1,1.2,1.3,1.5,2.0}.

	5^th^	10^th^	20^th^	30^th^	40^th^	50^th^	60^th^	70^th^	80^th^	90^th^	95^th^
*τ*	*n* = 5										
1.1	3	5	10	15	21	29	38	73	109	175	240
1.2	1	2	4	6	9	12	15	20	35	56	78
1.3	1	1	2	3	5	6	8	10	13	26	35
1.5	1	1	1	2	2	3	4	4	6	8	14
2.0	1	1	1	1	1	1	2	2	2	3	4
*τ*	*n* = 7										
1.1	2	4	8	13	18	24	32	57	86	139	191
1.2	1	2	3	5	7	9	12	15	23	41	57
1.3	1	1	2	3	3	5	6	8	10	18	25
1.5	1	1	1	1	2	2	3	3	4	6	9
2.0	1	1	1	1	1	1	1	1	2	2	3
*τ*	*n* = 10										
1.1	2	3	7	11	15	20	27	35	69	110	152
1.2	1	1	2	4	5	7	9	11	15	29	40
1.3	1	1	1	2	3	3	4	6	7	10	18
1.5	1	1	1	1	1	2	2	2	3	4	5
2.0	1	1	1	1	1	1	1	1	1	2	2
*τ*	*n* = 15										
1.1	2	3	6	9	12	16	21	28	52	83	115
1.2	1	1	2	3	4	5	6	8	11	20	27
1.3	1	1	1	1	2	2	3	4	5	7	11
1.5	1	1	1	1	1	1	1	2	2	3	4
2.0	1	1	1	1	1	1	1	1	1	1	1

Analysing the percentiles of the run length distribution will give us a better idea on the actual run length performance of the chart. For example, by referring to Table 2, when the optimal charting parameters for the *ARL*-based design is adopted, the *MRL*_0_ obtained is between 211 to 251, with smaller values obtained for smaller *τ* and *n*. This is significantly different from the *ARL*_0_ of 370.4, which shows that for 50% of the time, the false alarm will happen much earlier than the 370^th^ sample. When practitioners design the chart based on an *ARL*_0_ of 370.4, they would expect than most of the time, false alarms only happen by the 370^th^ sample. However, this is not the case from the *MRL*_0_ values obtained in Table 2. In fact, 370 falls between the 60^th^ and 70^th^ percentile. This shows the in-control run length distribution is skewed to the right, and interpreting the frequency of false alarms based purely on the *ARL*_0_ is not accurate. Furthermore, studying the difference between the extreme percentiles (for example, the difference between the 5^th^ and 95^th^ percentile) enables practitioners to have a better idea on the variation of the run length.

A similar trend is observed for the out-of-control percentiles, especially for small values of *τ*. In particular, there is a significant difference between the *ARL*_1_ in Table 1 with the *MRL*_1_ in Table 3. For example, for *n* = 5 and *τ* = 1.1, Table 3 shows an *MRL*_1_ of 29, but the *ARL*_1_ is 64.74 from Table 1. This shows that for more than 50% of the time, the out-of-control condition will be detected before what is indicated by the *ARL*_1_. In most cases, the *ARL*_1_ lies close to the 70^th^ percentile. Hence, evaluating the side sensitive synthetic-*γ* chart based purely on the *ARL*_1_ will result in an inaccurate representation of the actual performance of the chart when it is implemented since in most cases the shift is detected earlier than the *ARL*_1_.

From Table 3, the difference between the extreme percentiles reduces as *τ* and *n* increases. This shows that there is less variation in the out-of-control run lengths for larger *τ* and *n*. For example, for *n* = 5 and *τ* = 1.1, the difference between the 5^th^ and 95^th^ percentile is 237, while for *n* = 5 and *τ* = 2.0, the corresponding difference is only 3. Similarly, for *n* = 15 and *τ* = 1.1, the difference between the extreme percentiles is 113 (compared to the corresponding difference of 237 for *n* = 5 and *τ* = 1.1).

The percentiles in Tables 2 and 3 can only be obtained if *τ* can be specified in advance. Since *τ* cannot be specified in certain practical scenarios, this section also analyses the expected percentiles. In this paper, (*τ*_min_,*τ*_max_) is set as (1,2]. Table 4 shows the optimal charting parameters (*L*, *LCL* and *UCL*) and the *EARL* of the *EARL*-based design of the side sensitive synthetic-*γ* chart for *γ*_0_ = 0.05 and *n*∈{5,7,10,15}. Tables 5 and 6 show the in-control percentiles and out-of-control expected percentiles, respectively, when the charting parameters in Table 4 are adopted. The expected percentiles are obtained by substituting the optimal charting parameters in Table 4, together with the corresponding *γ*_0_, *n* and (*τ*_min_,*τ*_max_) = (1,2], into Eq (8).

**Table 4:** Optimal charting parameters and the corresponding *EARL* values for the *EARL*-based side sensitive synthetic-*γ* chart for *γ*_0_ = 0.05, *n*∈{5,7,10,15} and (*τ*_min_,*τ*_max_) = (1,2].

*n*	*L*	*LCL*	*UCL*	*EARL*
5	25	0.0036	0.0905	16.90
7	25	0.0128	0.0832	13.73
10	27	0.0199	0.0774	11.16
15	29	0.0260	0.0723	8.85

**Table 5:** In-control percentiles of the run length distribution for the *EARL*-based side sensitive synthetic-*γ* chart for *γ*_0_ = 0.05, *n*∈{5,7,10,15} and (*τ*_min_,*τ*_max_) = (1,2].

*n*	5^th^	10^th^	20^th^	30^th^	40^th^	50^th^	60^th^	70^th^	80^th^	90^th^	95^th^
5	5	10	21	71	140	222	323	452	635	947	1259
7	5	10	21	71	140	222	322	451	633	943	1254
10	5	10	22	70	139	219	318	446	625	932	1239
15	6	11	24	74	144	226	327	458	641	955	1268

**Table 6:** Out-of-control expected percentiles of the run length distribution for the *EARL*-based side sensitive synthetic-*γ* chart for *γ*_0_ = 0.05, *n*∈{5,7,10,15} and (*τ*_min_,*τ*_max_) = (1,2].

*n*	5^th^	10^th^	20^th^	30^th^	40^th^	50^th^	60^th^	70^th^	80^th^	90^th^	95^th^
5	1.24	1.65	2.56	3.82	5.84	9.02	13.58	18.85	27.82	43.04	57.82
7	1.15	1.49	2.29	3.32	4.96	7.33	10.90	15.56	22.63	34.49	47.04
10	1.15	1.39	2.15	2.87	4.07	6.06	8.71	12.61	18.02	27.81	37.37
15	1.12	1.36	1.90	2.59	3.24	4.76	7.14	9.83	14.63	22.05	29.92

The in-control percentiles in Table 5 show a similar trend as the *ARL*-based design. The *MRL*_0_ ranges from 219 to 226, which is significantly smaller than the *ARL*_0_ of 370.4. From Table 6, the *EMRL* is also significantly smaller than the *EARL*_1_. For example, for *n* = 5, the *EMRL* is 9.02, while the corresponding *EARL* is 16.90 from Table 4. The expected percentiles in Table 6 decreases for larger *n*. Similarly, the difference between the extreme expected percentiles decreases for larger *n*, which shows less variation in the expected percentiles for larger *n*.

## 4. *MRL* and *EMRL*-based design of the side sensitive synthetic-*γ* chart

As the *MRL* and *EMRL* provide more accurate results than the *ARL* and *EARL* when the run length distribution is skewed, this section proposes an alternative design where the optimal charting parameters which minimize the *MRL*_1_ and *EMRL* are obtained, subject to constraints in the *MRL*_0_.

The following are the algorithms to obtain the optimal charting parameters based on the *MRL*-based design.

1. Determine the values for *γ*_0_, *n* and *τ*.
2. Initialize *L* = 1.
3. Solve Eq (7) for *K* by setting *l*_0.5_ = *ξ* and *τ* = 1. Then, calculate *LCL* and *UCL* from Eqs (1) and (2), respectively. This combination of (*LCL*,*UCL*) will result in *MRL*_0_ = *ξ*.
4. By using the (*LCL*,*UCL*) in Step 3, numerically search for *l*_0.5_ that satisfies Eq (7) for the *γ*_0_, *n* and *τ* determined in Step 1. The *MRL*_1_ = *l*_0.5_.
5. Increase *L* by 1.
6. Repeat Steps 3 to 5 until the *MRL*_1_ for *L*+1 is larger than the *MRL*_1_ for *L*. This (*L*,*LCL*,*UCL*) combination will be the optimal charting parameters for the *MRL*-based side sensitive synthetic**-***γ* chart. If there is more than one combination of (*L*,*LCL*,*UCL*) with the smallest *MRL*_1_, the combination with the smallest value for the difference between *l*_0.95_ and *l*_0.05_ is chosen as the optimal charting parameters, where *l*_0.05_ and *l*_0.95_ are the 5^th^ and 95^th^ percentiles of the out-of-control run length distribution, respectively.

To ensure a fair comparison between the *ARL*-based and *MRL*-based design, *ξ* is set to be equivalent to the *MRL*_0_ in Table 2. For example, by referring to the *MRL*_0_ for *n* = 5 and *τ* = 1.1 in Table 2, *ξ* = 211. Table 7 shows the optimal charting parameters for the *MRL*-based design, and the 5^th^ percentile (Q05), *MRL*_1_, 95^th^ percentile (Q95), *ARL*_1_ and *ARL*_0_ when these optimal charting parameters are adopted are also shown. For example, for *n* = 5 and *τ* = 1.1, the optimal charting parameters for the *MRL*-based design are (*L*,*LCL*,*UCL*) = (22.0.0043,0.0898), and adopting these optimal charting parameters result in (*Q*05,*MRL*_1_,*Q*95,*ARL*_1_,*ARL*_0_) = (2.22,231,63.38,350.42).

**Table 7:** Optimal charting parameters and the corresponding Q05, *MRL*_1_, Q95, *ARL*_1_ and *ARL*_0_ for the *MRL*-based side sensitive synthetic-*γ* chart for *γ*_0_ = 0.05, *n*∈{5,7,10,15} and *τ*∈{1.1,1.2,1.3,1.5,2.0}.

***τ***	*n* = 5							
	*L*	*LCL*	*UCL*	Q05	*MRL*_1_	Q95	*ARL*_1_	*ARL*_0_
1.1	22	0.0043	0.0898	2	22	231	63.38	350.42
1.2	8	0.0083	0.0858	1	8	81	22.58	350.87
1.3	4	0.0111	0.0829	1	4	42	11.78	348.18
1.5	4	0.0110	0.0831	1	2	15	4.44	360.02
2.0	4	0.0109	0.0832	1	1	4	1.72	370.36
***τ***	*n* = 7							
	*L*	*LCL*	*UCL*	Q05	*MRL*_1_	Q95	*ARL*_1_	*ARL*_0_
1.1	18	0.0140	0.0820	2	18	186	51.10	344.30
1.2	6	0.0173	0.0787	1	6	62	17.42	350.38
1.3	3	0.0194	0.0766	1	3	31	8.93	350.55
1.5	8	0.0161	0.0798	1	2	8	3.04	375.27
2.0	3	0.0193	0.0767	1	1	3	1.38	366.77
***τ***	*n* = 10							
	*L*	*LCL*	*UCL*	Q05	*MRL*_1_	Q95	*ARL*_1_	*ARL*_0_
1.1	16	0.0212	0.0761	2	15	151	41.38	347.30
1.2	7	0.0231	0.0742	1	5	43	12.04	351.20
1.3	11	0.0219	0.0754	1	3	17	5.23	379.19
1.5	3	0.0251	0.0722	1	1	7	2.29	364.56
2.0	2	0.0260	0.0713	1	1	2	1.17	373.35
***τ***	*n* = 15							
	*L*	*LCL*	*UCL*	Q05	*MRL*_1_	Q95	*ARL*_1_	*ARL*_0_
1.1	13	0.0275	0.0708	1	12	117	32.12	353.27
1.2	3	0.0302	0.0681	1	3	34	9.64	356.54
1.3	9	0.0280	0.0702	1	2	9	3.55	369.80
1.5	3	0.0301	0.0681	1	1	3	1.63	368.30
2.0	1	0.0324	0.0659	1	1	1	1.08	363.63

By comparing the optimal charting parameters in Table 1 with that in Table 7, it can be observed that the optimal *L* for the *ARL*-based design is generally larger for small values of *τ* and *n* compared to the *MRL*-based design. For example, for *n* = 5 and *τ* = 1.1, the optimal *L* = 42 for the *ARL*-based design, while the optimal *L* = 22 for the *MRL*-based design. The smaller optimal *L* for the *MRL*-based design is also associated with a smaller conforming region, as shown by larger values of *LCL* and smaller values of *UCL*. For example, for *n* = 5 and *τ* = 1.1, the optimal (*LCL*, *UCL*) = (0.0017, 0.0924) for the *ARL*-based design, while the optimal (*LCL*, *UCL*) = (0.0043, 0.0898) for the *MRL* -based design.

Next, comparing the *MRL*_1_ in Table 3 with that in Table 7 shows that the *MRL*-based design results in smaller *MRL*_1_ compared with that in the *ARL*-based design, especially for small values of *τ*. For example, for *n* = 5 and *τ* = 1.1, the *MRL*_1_ = 29 for the *ARL*-based design in Table 3, while the *MRL*_1_ = 22 for the *ARL*-based design in Table 7. This shows that the *MRL*-based design results in better *MRL*_1_ performance compared with the *ARL*-based design. Note that both designs have the same *MRL*_0_.

Both the *MRL*-based and *ARL*-based designs show similar *ARL*_1_. For example, for *n* = 5 and *τ* = 1.1, the *ARL*_1_ = 64.74 for the *ARL*-based design in Table 1, while the *ARL*_1_ = 63.38 for the *MRL*-based design in Table 7. This shows that the *MRL*-based design results in a smaller median number of samples to detect the shift, but with a similar average number of samples to detect the shift.

The *ARL*_0_ is set as 370.4 for the *ARL*-based design, while the *ARL*_0_ for the *MRL*-based design in Table 7 is between 344.30 to 375.27. Note that the *MRL*-based design does not fix the value for *ARL*_0_. The *ARL*_0_ for the *MRL*-based design is generally smaller than that of the *ARL*-based design, however, the difference is not large.

The *MRL*-based design in Table 7 can only be implemented if *τ* can be specified in advance. This paper also considers the *EMRL*-based design for cases where *τ* is unknown. Similar steps as shown in paragraph 2 for the *MRL*-based design are adopted, but Steps 1, 4 and 6 are replaced with the following.

1 Determine the values for *γ*_0_, *n*, *τ*_min_ and *τ*_max_.
4 By using the (*LCL*,*UCL*) in Step 3, evaluate *E*(*l*_0.5_) from Eq (8). The *EMRL* = *E*(*l*_0.5_).
6 Repeat Steps 3 to 5 until the *EMRL* for *L*+1 is larger than the *EMRL* for *L*. This (*L*,*LCL*,*UCL*) combination will be the optimal charting parameters for the *EMRL*-based side sensitive synthetic**-***γ* chart.

Similar to the *EARL*-based design, (*τ*_min_,*τ*_max_) is set as (1,2]. To ensure a fair comparison between the *EARL*-based and *EMRL*-based design, *ξ* is set to be equivalent to the *MRL*_0_ in Table 5. Table 8 shows the optimal charting parameters for *n*∈{5,7,10,15} and its corresponding 5^th^ expected percentile (EQ05), *EMRL*, 95^th^ expected percentile (EQ95), *EARL* and *ARL*_0_ when these optimal charting parameters are adopted.

**Table 8:** Optimal charting parameters and the corresponding EQ05, *EMRL*, EQ95, *EARL* and *ARL*_0_ for the *EMRL*-based side sensitive synthetic-*γ* chart for *γ*_0_ = 0.05, *n*∈{5,7,10,15} and (*τ*_min_,*τ*_max_) = (1,2].

*n*	*L*	*LCL*	*UCL*	EQ05	*EMRL*	EQ95	*EARL*	*ARL*_*0*_
5	7	0.0089	0.0852	1.10	9.90	57.99	17.20	344.46
7	15	0.0144	0.0816	1.12	7.49	46.17	13.58	356.84
10	14	0.0215	0.0758	1.12	5.90	37.05	11.02	349.06
15	12	0.0276	0.0706	1.05	5.03	29.72	8.97	354.87

By comparing the optimal charting parameters for the *EMRL*-based design in Table 8 with the corresponding optimal charting parameters for the *EARL*-based design in Table 4, it can be observed that the optimal *L* in Table 8 is smaller than the optimal *L* in Table 4. Furthermore, the conforming region for the *EMRL*-based design is smaller than that of the *EARL*-based design, as shown by the larger *LCL* and smaller *UCL* in Table 8, compared to the *LCL* and *UCL* in Table 4. Minimal differences are shown between the EQ05, *EMRL* and EQ95 values of the *EARL* and *EMRL*-based designs, by comparing Tables 6 and 8. The *EARL* values in Tables 4 and 8 are also similar. Hence, the *EARL* and *EMRL*-based designs show similar performance. The *ARL*_0_ of the *EMRL*-based design is slightly smaller than the *ARL*_0_ of the *EARL*-based design, but the difference is not very large. Note that the *ARL*_0_ of the *EARL*-based design is fixed as 370.4.

## 5. Comparisons

In this section, the *MRL* and *EMRL*-based designs of the side sensitive synthetic-*γ* chart is compared with the corresponding designs for the synthetic-*γ*chart without side sensitivity. To obtain the *MRL* and *EMRL*-based designs for the synthetic-*γ* chart without side sensitivity, a similar procedure as shown in Section 4 is adopted, but modified for the synthetic-*γ* chart without side sensitivity. Table 9 shows the Q05, *MRL*_1_ and Q95 for the *MRL*-based designs of these two charts, while Table 10 shows the EQ05, *EMRL*_1_ and EQ95 for the *EMRL*-based designs.

**Table 9:** Comparison of the Q05, *MRL*_1_ and Q95 of the *MRL*-based synthetic-*γ* chart (without side sensitivity) and side sensitive synthetic-*γ* chart for *γ*_0_ = 0.05, *n*∈{5,7,10,15} and *τ*∈{1.1,1.2,1.3,1.5,2.0}.

*τ*	*n* = 5					
	Synthetic-*γ* Chart			Side Sensitive Synthetic-*γ* Chart		
	Q05	*MRL*_1_	Q95	Q05	*MRL*_1_	Q95
1.1	3	32	343	2	22	231
1.2	1	10	106	1	8	81
1.3	1	5	52	1	4	42
1.5	1	2	23	1	2	15
2.0	1	1	4	1	1	4
*τ*	*n* = 7					
	Synthetic-*γ* Chart			Side Sensitive Synthetic-*γ* Chart		
	Q05	*MRL*_1_	Q95	Q05	*MRL*_1_	Q95
1.1	2	27	279	2	18	186
1.2	1	8	85	1	6	62
1.3	1	4	40	1	3	31
1.5	1	2	15	1	2	8
2.0	1	1	3	1	1	3
*τ*	*n* = 10					
	Synthetic-*γ* Chart			Side Sensitive Synthetic-*γ* Chart		
	Q05	*MRL*_1_	Q95	Q05	*MRL*_1_	Q95
1.1	2	23	236	2	15	151
1.2	1	7	64	1	5	43
1.3	1	3	27	1	3	17
1.5	1	1	13	1	1	7
2.0	1	1	2	1	1	2
*τ*	*n* = 15					
	Synthetic-*γ* Chart			Side Sensitive Synthetic-*γ* Chart		
	Q05	*MRL*_1_	Q95	Q05	*MRL*_1_	Q95
1.1	2	18	189	1	12	117
1.2	1	5	45	1	3	34
1.3	1	2	18	1	2	9
1.5	1	1	4	1	1	3
2.0	1	1	1	1	1	1

**Table 10:** Comparison of the EQ05, *EMRL*_1_ and EQ95 of the *EMRL*-based synthetic-*γ* chart (without side sensitivity) and side sensitive synthetic-*γ* chart for *γ*_0_ = 0.05, *n*∈{5,7,10,15} and (*τ*_min_,*τ*_max_) = (1,2].

*n*	Synthetic-*γ* Chart			Side Sensitive Synthetic-*γ* Chart		
	EQ05	*EMRL*_1_	EQ95	EQ05	*EMRL*_1_	EQ95
5	1.24	12.98	79.49	1.10	9.90	57.99
7	1.17	11.05	67.49	1.12	7.49	46.17
10	1.15	10.07	58.92	1.12	5.90	37.05
15	1.17	7.71	47.52	1.05	5.03	29.72

From Table 9, the side sensitive synthetic-*γ* chart shows smaller *MRL*_1_ and Q95 compared to the synthetic-*γ* chart without side sensitivity, especially for small values of *τ*. For example, for *n* = 5 and *τ* = 1.1, (*MRL*_1_,Q95) = (32,343) for the synthetic-*γ* chart without side sensitivity, whereas (*MRL*_1_,Q95) = (22,231) for the side sensitive synthetic-*γ* chart. This shows that incorporating side sensitivity reduces the median number of samples required to detect the shift, and at the same time reduces the variability in the run length due to a smaller difference between Q95 and Q05. This is consistent with the results by Yeong et al. (2021), which shows that the side sensitive synthetic-*γ* chart shows better *ARL* performance than the synthetic-*γ* chart without side sensitivity. From Table 10, a similar conclusion is reached for the *EMRL*-based design. Thus, it can be concluded that the side sensitive synthetic-*γ* chart shows better performance than the synthetic-*γ* chart without side sensitivity.

## 6. Illustrative example

This section shows the implementation of the *MRL* and *EMRL*-based design on an actual industrial example. The example was also adopted by Yeong et al. [24] who proposed the side sensitive synthetic-*γ* chart. The example is from a sintering process where compressed metal powder is heated to a temperature that allows bonding of the individual particles. The strength of the bond between particles is influenced by pore shrinkage [39]. One of the characteristics that is related to pore shrinkage is the pressure test drop time (*T*_pd_) from 2 bar to 1.5 bar, which must be larger than 30 seconds.

Sintering steel with a heterogeneous microstructure and an irregular grain size will lead to an anomalous increase in the standard deviation of *T*_pd_ (*σ*_pd_), which will result in a change in the correlation structure between the mean of *T*_pd_ (*μ*_pd_) and *σ*_pd_ and subsequently results in shifts in the coefficient of variation of *T*_pd_ (*γ*_pd_). Hence, the special cause can be detected by monitoring *γ*_pd_. Furthermore, Castagliola et al. [6] through a regression study showed that *σ*_pd_ = *γ*_pd_×*μ*_pd_, which provides additional evidence that the process can be monitored by detecting changes in *γ*_pd_.

Table 11 (left) shows a Phase I dataset of *m* = 20 samples, each with a sample size *n* = 5. ${\bar{X}}_{k},S_{k}$ and ${\hat{\gamma }}_{k}$ denotes the sample mean, standard deviation and coefficient of variation, respectively, of the *k*^th^ sample, *k* = 1, 2, … 20. The data is taken from Castagliola et al. [6]. Castagliola et al. [6] showed that the Phase I data were in-control, with an estimated in-control *γ* $\left({\hat{\gamma }}_{0}\right)$ of 0.417 based on a root-mean-square computation.

**Table 11:** Phase I and Phase II datasets from a sintering process.

Phase I				Phase II			
*k*	${\bar{X}}_{k}$	*S*_*k*_	${\hat{\gamma }}_{k}$	*k*	${\bar{X}}_{k}$	*S*_*k*_	${\hat{\gamma }}_{k}$
1	664.2	268.9	0.405	1	906.4	476.0	0.525
2	705.6	308.6	0.437	2	805.1	493.9	0.613
3	1051.5	539.9	0.513	3	1187.2	1105.9	0.932
4	1047.3	359.0	0.343	4	663.4	304.8	0.459
5	618.2	136.3	0.220	5	1012.1	367.4	0.363
6	781.4	446.4	0.571	6	863.2	350.4	0.406
7	797.8	342.5	0.429	7	1561.0	1652.2	1.058
8	678.9	275.4	0.406	8	697.1	253.2	0.363
9	848.3	320.5	0.378	9	1024.6	120.9	0.118
10	1015.3	453.7	0.447	10	355.3	235.2	0.662
11	777.4	276.4	0.356	11	485.6	106.5	0.219
12	813.9	170.7	0.210	12	1224.3	915.4	0.748
13	716.9	397.4	0.554	13	1365.0	1051.6	0.770
14	937.6	421.2	0.449	14	704.0	449.7	0.639
15	915.1	331.9	0.363	15	1584.7	1050.8	0.663
16	873.2	285.0	0.326	16	1130.0	680.6	0.602
17	984.3	573.7	0.583	17	824.7	393.5	0.477
18	819.3	156.2	0.191	18	921.2	391.0	0.424
19	839.0	244.0	0.291	19	870.3	730.0	0.839
20	585.8	322.3	0.550	20	1068.3	150.8	0.141

**Fig 2:**
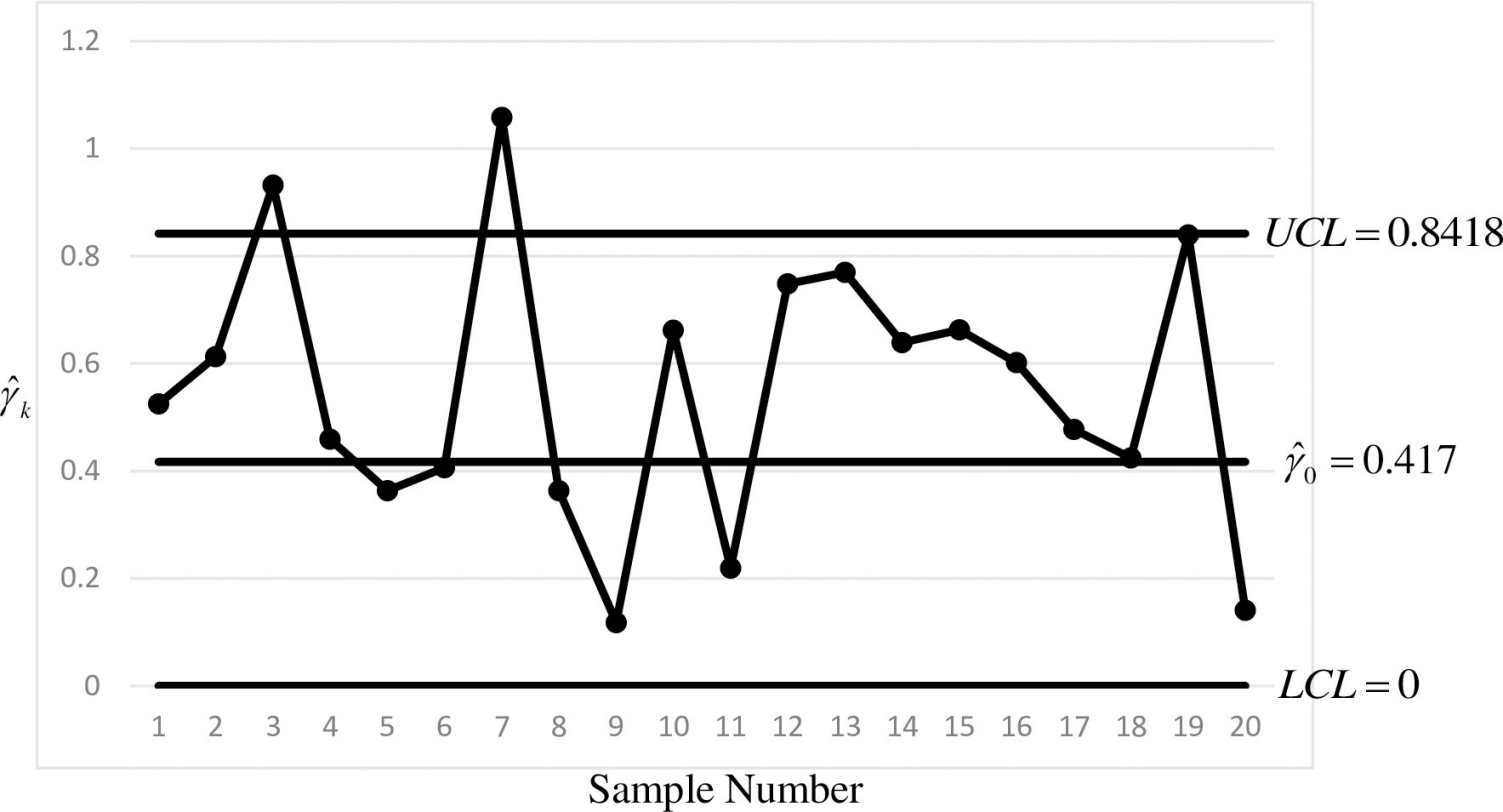
The *γ* sub-chart of the *MRL*-based side-sensitive synthetic-*γ* chart applied to the sintering process (Phase II).

Table 11 (right) shows the Phase II data after the occurrence of a special cause that increased process variability. A shift of 25% in the coefficient of variation shows that something is wrong in the production of the parts. Hence, the side-sensitive synthetic-*γ* chart is designed to detect a shift of *τ* = 1.25. The *MRL*-based design in Section 4 is adopted to obtain the optimal charting parameters, where *ξ* = 250. The optimal charting parameters are (*L*,*LCL*,*UCL*) = (7,0,0.8418), which results in (Q05,*MRL*_1_,Q95) = (1,7,76). By comparison, the *ARL*-based design by Yeong et al. [24] results in (Q05,*MRL*_1_,Q95) = (1,10,68). Fig 2 shows the *γ* sub-chart of the side-sensitive synthetic-*γ* chart by adopting the *MRL*-based design.

From Fig 2, two non-conforming samples are detected, i.e. Samples 3 and 7, with *CRL* of 3 and 4, respectively. Since both *CRL*s are less than 7, out-of-control signals are produced at Samples 3 and 7. This shows that the side-sensitive synthetic-*γ* chart can show a quick detection of the special cause. The *ARL*-based design by Yeong et al. [24] also detected the out-of-control condition at Samples 3 and 7.

**Fig 3:**
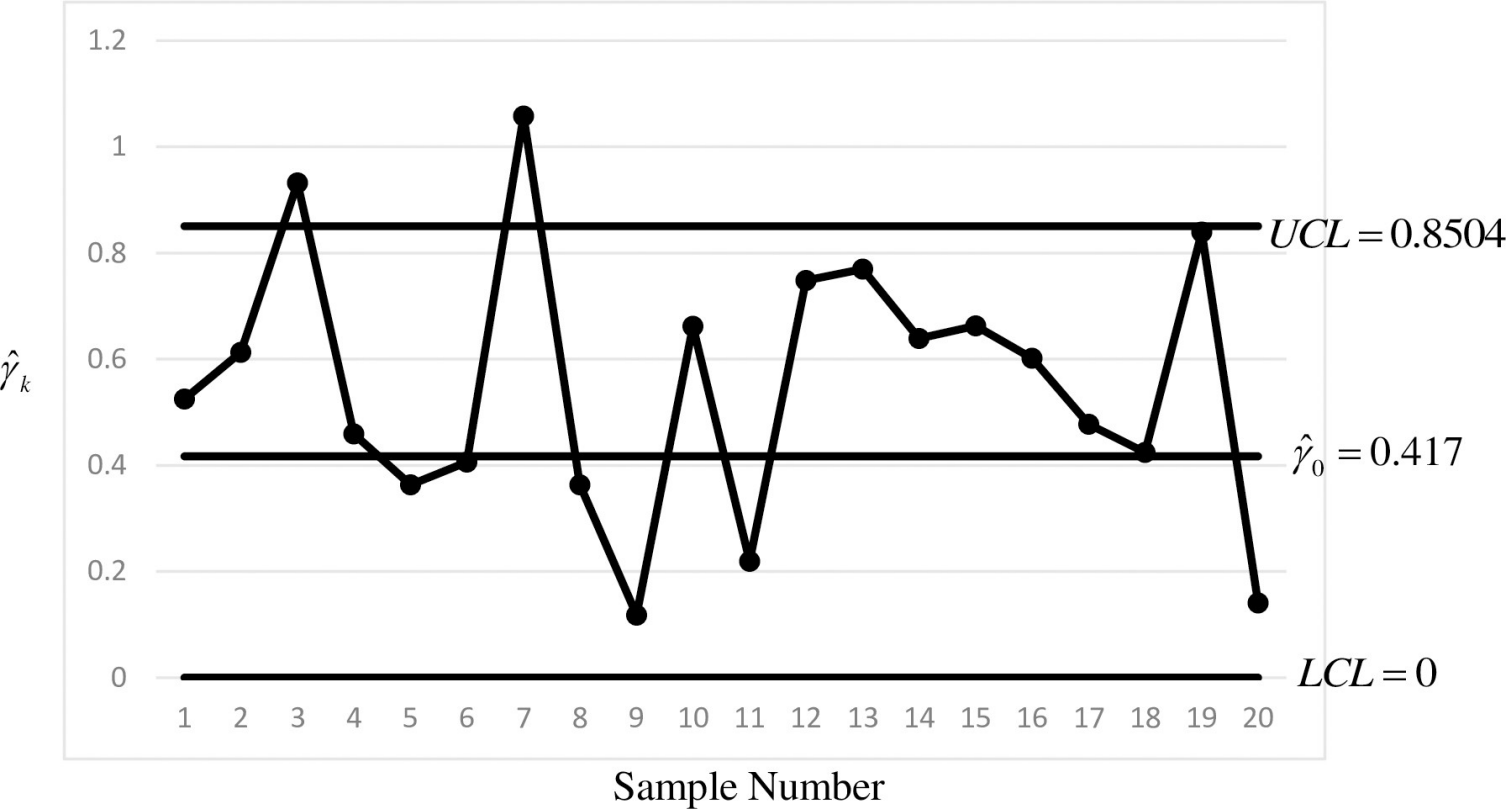
The *γ* sub-chart of the *EMRL*-based side-sensitive synthetic-*γ* chart applied to the sintering process (Phase II).

The *MRL*-based design can only be adopted if *τ* can be specified. As the exact size of shift caused by the special cause may not be known, this section also considers the *EMRL*-based design with (*τ*_min_,*τ*_max_) = (1,2]. From the *EMRL*-based design in Section 4, the optimal charting parameters are (*L*,*LCL*,*UCL*) = (8,0,0.8504), which results in (EQ05,*EMRL*_1_,EQ95) = (1.12,12.43,74.94). Note that as in the *MRL*-based design, *ξ* = 250. Fig 3 shows the *γ* sub-chart of the side-sensitive synthetic-*γ* chart by adopting the *EMRL*-based design. Similar to the *MRL*-based design, Samples 3 and 7 are detected as out-of-control samples.

## 7. Conclusions

This paper evaluates the performance of the side-sensitive synthetic-*γ* chart by studying its’ entire run length distribution. An analysis of the run length distribution shows that false alarms frequently happens much earlier than that indicated by the *ARL*_0_. In addition, small shifts are often detected earlier compared to the *ARL*_1_. This will reduce the confidence towards the chart when the actual performance is frequently different from that indicated by the *ARL*. Hence, the performance of the side-sensitive synthetic-*γ* chart should not be evaluated only in terms of the *ARL*, but should be accompanied by the entire run length distribution. This paper also proposes alternative designs for the side-sensitive synthetic-*γ* chart based on the *MRL* and *EMRL*. Compared to designs based on the *ARL*, the proposed design based on the *MRL* shows better *MRL*_1_ performance, i.e. it requires a smaller median number of samples to detect shifts in *γ*. For the proposed design based on the *EMRL*, the performance is similar to that based on the *EARL*. Comparisons with the synthetic-*γ* chart without side sensitivity shows that side sensitivity reduces the median number of samples required to detect a shift and reduces the variability in the run length. Implementation of the proposed designs on an actual industrial example shows that they are efficient in detecting out-of-control conditions.

## Supporting information

S1 Appendix (DOCX)
