# Correction: Optimal designs of the side sensitive synthetic chart for the coefficient of variation based on the median run length and expected median run length

**DOI:** 10.1371/journal.pone.0271855

**Published:** 2022-07-18

**Authors:** Wai Chung Yeong, Ping Yin Lee, Sok Li Lim, Peh Sang Ng, Khai Wah Khaw

The first author’s name is spelled incorrectly. The correct name is: Wai Chung Yeong.
