# Correction: Optimal designs of the side sensitive synthetic chart for the coefficient of variation based on the median run length and expected median run length

**DOI:** 10.1371/journal.pone.0315118

**Published:** 2024-12-03

**Authors:** Waie Chung Yeong, Ping Yin Lee, Sok Li Lim, Peh Sang Ng, Khai Wah Khaw

There are errors in the Funding statement. The correct Funding statement is as follows: This research is supported by the Ministry of Higher Education Malaysia, in the form of a Fundamental Research Grant Scheme (FRGS), project code FRGS/1/2018/STG06/UM/02/3 (FP052-2018A) awarded to SLL and Sunway University in the form of an Individual Research Grant awarded to WCY (GRTIN-IRG-69-2021).
