# Supplementary material for: Optimal designs of the side sensitive synthetic chart for the coefficient of variation based on the median run length and expected median run length
Source: PLoS One. 2021 Jul 30;16(7):e0255366. doi: 10.1371/journal.pone.0255366 (PMC8323885; doi:10.1371/journal.pone.0255366)
Supplement: S1 Appendix — (DOCX) [file pone.0255366.s001.docx]

**S1 Appendix**

This section shows the formulae to evaluate the *ARL*, *SDRL* and *EARL* of the side sensitive synthetic-$\gamma$ chart through a Markov chain approach. Let 0 denote a sample falling between the *LCL* and *UCL*, 1 denote a sample falling below the *LCL* and 1 denote a sample falling above the *UCL*. The states of the Markov chain are defined as follows:

State 1 : 100..0

State 2 : 010..0

State 3 : 001..0

State *L* : 000..1

State *L* + 1 : 00..00

State *L* + 2 : 0..001

State *L* + 3 : 0..010

State 2*L* : 010..0

State 2*L* + 1: 100..0

State 2*L* + 2: Signaling state (i.e. the state where the chart signals an out-of-control condition)

$\begin{matrix} \text{ }\begin{matrix} 1 & \text{ }2 & \text{ }.. & L-1 & \text{ }L & \text{ }L+1 & L+2 & L+3 & L+4 & .. & 2L+1 & 2L+2 \end{matrix} \\ \mathbf{P}=\left(\begin{matrix} \mathbf{Q} & \mathbf{r} \\ \mathbf{0}^{T} & 1 \end{matrix}\right)=\begin{matrix} 1 \\ 2 \\ 3 \\ : \\ L \\ L+1 \\ L+2 \\ L+3 \\ : \\ 2L \\ 2L+1 \\ 2L+2 \end{matrix}\left(\begin{matrix} 0 & 0 & .. & \text{ }0 & \text{ 0 } & \text{ }A & \text{ }B^{+} & \text{ }0 & \text{ 0} & \text{ }.. & \text{ }0 & \text{ }B^{-} \\ A & 0 & .. & \text{ }0 & \text{ 0 } & \text{ }0 & \text{ }B^{+} & \text{ }0 & \text{ }0 & \text{ }.. & \text{ }.. & \text{ }B^{-} \\ 0 & A & .. & \text{ }0 & \text{ }0 & \text{ }0 & \text{ }B^{+} & \text{ }0 & \text{ }0 & \text{ }.. & \text{ }.. & \text{ }B^{-} \\ .. & .. & .. & \text{ }.. & \text{ }.. & \text{ }.. & .. & \text{ }.. & \text{ }.. & \text{ }.. & \text{ }.. & \text{ }.. \\ 0 & 0 & .. & \text{ }A & \text{ }0 & \text{ }0 & \text{ }B^{+} & \text{ }0 & \text{ }0 & \text{ }.. & \text{ }0 & \text{ }B^{-} \\ 0 & 0 & .. & 0 & \text{ }B^{-} & \text{ }A & B^{+} & \text{ }0 & \text{ }0 & \text{ }.. & \text{ }.. & 0 \\ 0 & 0 & .. & 0 & \text{ }B^{-} & \text{ 0} & 0 & \text{ }A & \text{ }0 & \text{ }.. & \text{ }0 & B^{+} \\ 0 & 0 & .. & 0 & B^{-} & \text{ }0 & 0 & \text{ 0} & \text{ }A & \text{ }.. & \text{ }0 & B^{+} \\ .. & .. & .. & .. & .. & \text{ }.. & .. & \text{ }.. & \text{ }.. & \text{ }.. & \text{ }.. & .. \\ 0 & 0 & .. & 0 & B^{-} & \text{ }0 & 0 & \text{ }0 & \text{ }0 & \text{ }.. & \text{ }A & B^{+} \\ 0 & 0 & .. & 0 & B^{-} & A & 0 & \text{ }0 & \text{ }0 & \text{ }0 & \text{ }0 & B^{+} \\ 0 & 0 & .. & 0 & 0 & 0 & 0 & \text{ }0 & \text{ }0 & \text{ }0 & \text{ }0 & 1 \end{matrix}\right) \end{matrix}$A $\left(2L+2\right)\times\left(2L+2\right)$ transition probability matrix based on the states defined in the preceding paragraph can be formed as follows:

(A1)

where

$$A=P\left(LCL<\hat{\gamma}<UCL\right)$$

$$=F_{\hat{\gamma}}\left(\left.UCL\right|n,\gamma\right)-F_{\hat{\gamma}}\left(\left.LCL\right|n,\gamma\right)$$

$=F_{t}\left(\left.\frac{\sqrt{n}}{LCL}\right|n-1,\frac{\sqrt{n}}{\gamma}\right)-F_{t}\left(\left.\frac{\sqrt{n}}{UCL}\right|n-1,\frac{\sqrt{n}}{\gamma}\right)$, (A2)

$$B^{+}=P\left(\hat{\gamma}>UCL\right)$$

$$=1-F_{\hat{\gamma}}\left(\left.UCL\right|n,\gamma\right)$$

$=F_{t}\left(\left.\frac{\sqrt{n}}{UCL}\right|n-1,\frac{\sqrt{n}}{\gamma}\right)$, (A3)

$$B^{-}=P\left(\hat{\gamma}<LCL\right)$$

$$=F_{\hat{\gamma}}\left(\left.LCL\right|n,\gamma\right)$$

$=1-F_{t}\left(\left.\frac{\sqrt{n}}{LCL}\right|n-1,\frac{\sqrt{n}}{\gamma}\right)$, (A4)

where $F_{t}\left(.\left|n-1,\frac{\sqrt{n}}{\gamma}\right.\right)$ is the cumulative distribution function (c.d.f.) of the non-central *t*-distribution with $\left(n-1\right)$ degrees of freedom and non-centrality parameter $\frac{\sqrt{n}}{\gamma}$.

The *ARL* and *SDRL* can be computed by adopting the factorial moments for the number of steps until the process reaches the absorbing state (State $\left(2L+2\right)$). By adopting the factorial moments by Latouche and Ramaswami [37], the *ARL* and *SDRL* can be computed as

$ARL=\mathbf{q}^{T}\left(\mathbf{I}-\mathbf{Q}\right)^{-1}\mathbf{1}$ (A5)

and

$SDRL=\sqrt{2\mathbf{q}^{T}\left(\mathbf{I}-\mathbf{Q}\right)^{-2}\mathbf{Q1}-ARL^{2}+ARL}$, (A6)

where $\mathbf{q}$ is the $\left(2L+1\right)\times1$ vector of initial probabilities associated with the transient states, $\mathbf{I}$ is an identity matrix, and $\mathbf{1}$ is a vector of ones. In this paper, a zero-state condition is assumed, thus, the $\left(L+2\right)^{\text{th}}$ element of $\mathbf{q}$ is one, while all other elements of $\mathbf{q}$ are zeros. To calculate the $ARL_{1}$ and out-of-control *SDRL* ($SDRL_{1}$), $\gamma=\tau\gamma_{0}$ is substituted into Equations (A5) and (A6), while to compute the $ARL_{0}$ and in-control *SDRL* ($SDRL_{0}$), $\gamma=\gamma_{0}$ is substituted into Equations (A5) and (A6). Note that $\tau$ is the shift size.

The *EARL* can be computed as follows:

$EARL=\int_{\tau_{\min}}^{\tau_{\max}} f_{\tau}\left(\tau\right)ARL\left(\tau,\gamma_{0},n,K\right)d\tau$ (A7)

where $f_{\tau}\left(\tau\right)$ is the probability density function (p.d.f.) of $\tau$ that is assumed to be uniformly distributed over $\left(\tau_{\min},\tau_{\max}\right)$. The Gauss-Legendre quadrature is adopted to solve the integral [38].
